# The time-course of distractor-based activation modulates effects of speed-accuracy tradeoffs in conflict tasks

**DOI:** 10.3758/s13423-021-02003-x

**Published:** 2021-12-16

**Authors:** Victor Mittelstädt, Jeff Miller, Hartmut Leuthold, Ian Grant Mackenzie, Rolf Ulrich

**Affiliations:** 1 Department of Psychology, University of Tübingen, Schleichstraße 4, 72076 Tübingen, Germany; 2 Department of Psychology, University of Otago, PO Box 56, Dunedin, 9054 New Zealand

**Keywords:** Simon effect, Eriksen flanker effect, Speed-accuracy tradeoff, Conflict tasks, Time pressure, Delta plots

## Abstract

The cognitive processes underlying the ability of human performers to trade speed for accuracy is often conceptualized within evidence accumulation models, but it is not yet clear whether and how these models can account for decision-making in the presence of various sources of conflicting information. In the present study, we provide evidence that speed-accuracy tradeoffs (SATs) can have opposing effects on performance across two different conflict tasks. Specifically, in a single preregistered experiment, the mean reaction time (RT) congruency effect in the Simon task increased, whereas the mean RT congruency effect in the Eriksen task decreased, when the focus was put on response speed versus accuracy. Critically, distributional RT analyses revealed distinct delta plot patterns across tasks, thus indicating that the unfolding of distractor-based response activation in time is sufficient to explain the opposing pattern of congruency effects. In addition, a recent evidence accumulation model with the notion of time-varying conflicting information was successfully fitted to the experimental data. These fits revealed task-specific time-courses of distractor-based activation and suggested that time pressure substantially decreases decision boundaries in addition to reducing the duration of non-decision processes and the rate of evidence accumulation. Overall, the present results suggest that time pressure can have multiple effects in decision-making under conflict, but that strategic adjustments of decision boundaries in conjunction with different time-courses of distractor-based activation can produce counteracting effects on task performance with different types of distracting sources of information.

## Introduction

One of the most basic characteristics of the human cognitive system is the ability to trade off speed for accuracy in decision-making (e.g., Bogacz et al., 2006; Heitz, 2014; Luce, 1986; Pachella, 1974). Specifically, results from a variety of different perceptual decision-making tasks have shown that task processing time and accuracy jointly increase or decrease (e.g., Khodadadi et al., 2017; Miller et al., 2008; Rae et al., 2014; Steinemann et al., 2018). Investigating the underlying mechanisms of this joint function, the so-called speed-accuracy tradeoff (SAT), is a continuing concern within the field of cognitive psychology. The present study aims to contribute to this investigation by demonstrating that SATs can differentially affect task performance (i.e., reaction time (RT)) when making decisions in the presence of conflicting sources of information (e.g., Eriksen & Eriksen, 1974; Simon & Rudell, 1967). Moreover, we show that the somewhat paradoxical empirical finding of increased versus decreased mean congruency RT effects in Simon versus Eriksen tasks with time pressure can be explained within processing architectures that incorporate the idea of time-based processing of distracting information (i.e., location and flankers) by additionally examining the corresponding distributional RT patterns and simulating and fitting the data to the Diffusion Model for Conflict (DMC) tasks (Ulrich et al., 2015).

Probably the most widely used approach to study SATs is to manipulate time demands (e.g., by means of instructions) in two-choice RT tasks and to describe the potential underlying processes within the architecture of evidence accumulation models (e.g., Bogacz et al., 2006; Ratcliff & McKoon, 2008). In their most basic form, these models propose that a single, noisy decision-making process accumulates evidence with a certain rate (i.e., drift rate). As soon as a criterion amount of evidence needed to select a response is reached (i.e., one of two decision boundaries), a response is executed (for recent reviews, see, e.g., Evans & Wagenmakers, 2020; Ratcliff et al., 2016). These models can account for SATs in a straightforward and intuitive way by assuming changes in the height of decision boundaries: Higher decision boundaries lead to slower but more accurate decisions, whereas lower decision boundaries lead to faster but less accurate decisions (e.g., Bogacz et al., 2006; Lerche & Voss, 2018; Ratcliff & McKoon, 2008). Interestingly, however, recent findings suggest that this standard account may sometimes be incomplete (e.g., Kloosterman et al., 2019; Rae et al., 2014; Steinemann et al., 2018). For example, the externally specified available processing time might also affect the rate of evidence accumulation towards the correct decision boundary (e.g., Rae et al., 2014; Servant et al., 2019). Furthermore, processes attributed to the non-decision time might be sensitive to temporal demands – that is, processes after the start of motor activation of the selected response (e.g., Lerche & Voss, 2018; Osman et al., 2000; Rinkenauer et al., 2004; Spieser et al., 2017).

Critically, over the past two decades, researchers have shown an increased interest in investigating the nature of SATs in experimental paradigms where people select responses in the presence of multiple sources of information, both relevant (i.e., targets) and irrelevant (i.e., distractors) (e.g., Dambacher & Hübner, 2015; Hedge et al., 2019; Spieser et al., 2017; Van der Lubbe et al., 2001; Van Veen et al., 2008; Wylie et al., 2009). Two of these paradigms are particularly relevant for the present study: First, in the standard visual Simon task, a stimulus (e.g., a letter S or H) is presented to the left or right of fixation on the computer screen, but participants are required to ignore the task-irrelevant stimulus location and to make a left or right response on the basis of task-relevant non-spatial stimulus information (e.g., pressing a left key when the letter is an S or a right key when the letter is an H; Simon, 1990). Second, in the standard Eriksen flanker task, the stimulus containing the task-relevant response information is presented at the center of the screen in each trial (e.g., a letter S or H), but the target stimulus is flanked on each side by response-congruent or -incongruent task-irrelevant letters (Eriksen & Eriksen, 1974). In the Simon task, RTs are faster when stimulus location and task-relevant response are on the same side (i.e., congruent trials) compared to when they are on opposite sides (incongruent trials) and such congruency effects are also observed in the Eriksen task with faster RTs when the flankers are congruent (e.g., SSSSS) than when they are incongruent (e.g., SSHSS) with the target letter and hence the required response.

The specific goals of the above-mentioned SAT-Simon and SAT-Eriksen task studies differed in many respects, but the presence of SATs in all of these studies is at least partially explained by adjustments of decision boundaries (e.g., Dambacher & Hübner, 2015; Hedge et al., 2019; Van Veen et al., 2008). Furthermore, motor processes seem to be affected by SAT adjustments in both Simon (Servant et al., 2018; Van der Lubbe et al., 2001) as well as Eriksen task studies (e.g., Rinkenauer et al., 2004; Spieser et al., 2017). However, we noticed some hints that point to a crucial discrepancy when reviewing the effects of SAT manipulations on the congruency RT effects.^1^ Although congruency effects were present in both Simon and Eriksen SAT-conflict paradigms, the Eriksen congruency effect was typically larger with accuracy compared to speed focus (e.g., Dambacher & Hübner, 2015; Hedge et al., 2019; Spieser et al., 2017; Wylie et al., 2009), whereas the Simon congruency effect was larger with speed compared to accuracy focus (e.g., Van der Lubbe et al., 2001; Van Veen et al., 2008). However, this observation has neither been discussed nor directly tested within a single experiment designed for that purpose. The primary goal of the present study was to directly test the hypothesis that speed pressure can differentially affect task performance with different sources of distracting information (i.e., irrelevant flankers vs. irrelevant location). Thus, participants were required to make choice responses to the same target letters in Simon and Eriksen tasks that alternated from block to block, and task variation was combined with an SAT manipulation.

Another goal of the present study was to understand why SATs can have potentially opposing effects on performance across these two tasks. On the one hand, such differential changes in congruency effects with speed versus accuracy instructions would seem puzzling when considering that congruency effects are usually explained by dual-route models according to which activation produced by task-irrelevant distracting features superimposes with activation produced by task-relevant target features during decision-making (e.g., De Jong et al., 1994; Eimer et al., 1995; Logan, 1980; Posner & Snyder, 1975). Thus, reconciling this idea with the finding of different SAT effects on the two congruency effects would imply additional assumptions about differences between the two tasks. For example, perhaps (a) distractor-based activation is superimposed with task-relevant processes at different processing levels in the two conflict tasks, and/or (b) the effects of SAT manipulations differ somewhat between the two conflict tasks.

**Fig. 1:**
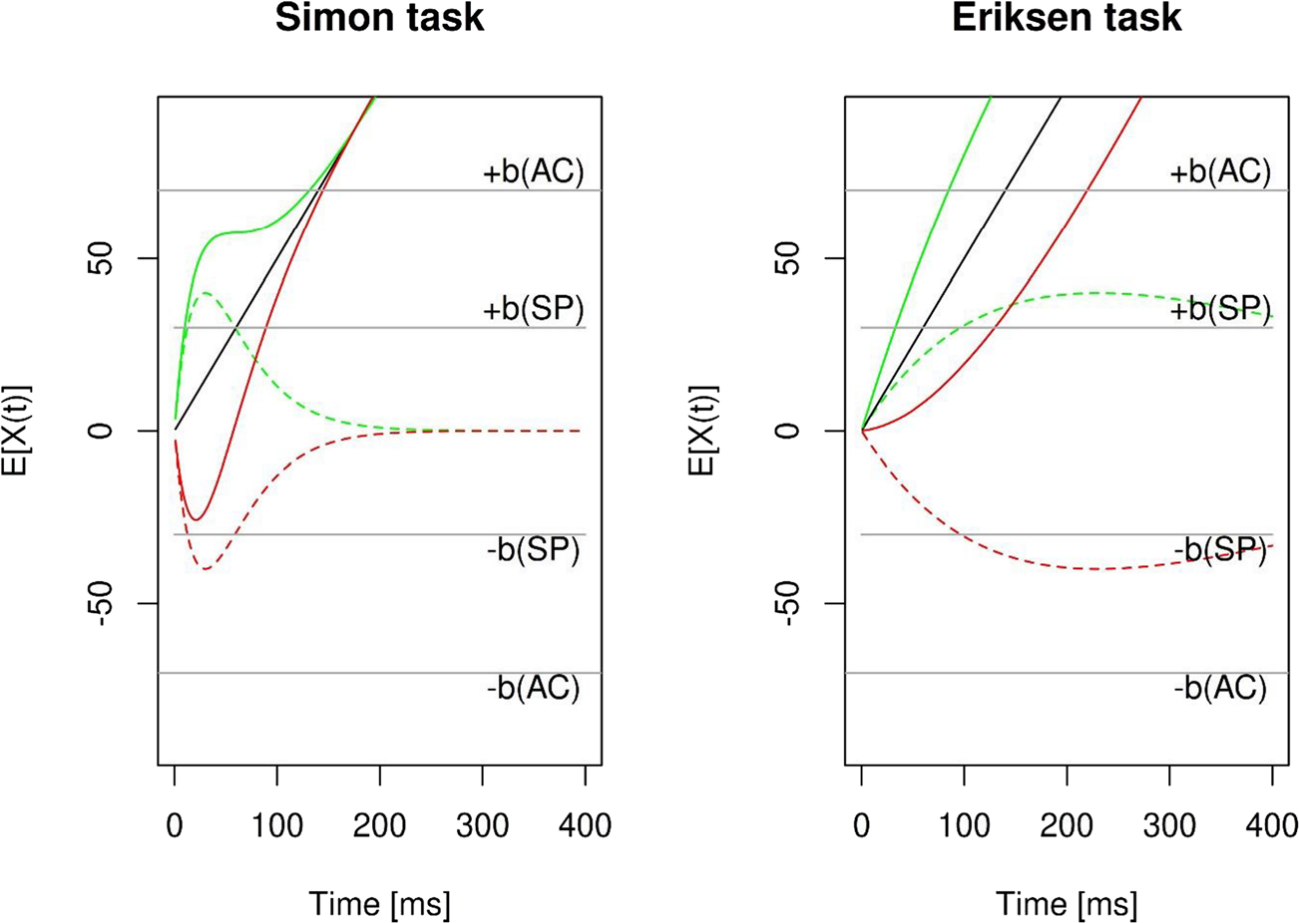
Mean activation functions based on simulation results of the Diffusion Model for Conflict (Ulrich et al., 2015) tasks using the R-package provided by Mackenzie and Dudschig (2021). For each condition, 100,000 trials with a stepsize of t = 1 ms were simulated. Except as described otherwise, the same model parameters were used that are displayed in Table 1 by Ulrich et al. (2015). Solid black lines depict target-based activations, dotted colored lines depict congruent (green) and incongruent (red) distractor-based activations, solid colored lines depict the superimposed activations in the congruent (green) and incongruent (red) condition, grey lines depict upper and lower decision boundaries in the speed (*b*[*SP*] = 30) and accuracy (*b*[*AC*] = 70) condition, respectively. Following Ulrich et al. (2015) the parameters were: *a* = 2, *σ* = 4, *μ*_c_ =0.5, *μ*_R_ = 300 and *σ*_R_ =30. For the Simon task, the amplitude of the distractor-based activation was *A* = 40 and already maximal at *τ* = 30 ms. For the Eriksen task, the amplitude of the distractor-based activation was *A* = 40 and maximal at *τ* = 230 ms

On the other hand, differential SAT influences on Simon versus Eriksen flanker effects could also be reconciled without assuming different cognitive mechanisms operating across conflict tasks and by just relying on the standard idea of varying decision boundaries to account for the effects of the SAT manipulations. Specifically, one has to consider that distractor-based processes may just unfold differently in time across different conflict tasks, as has been suggested within a recent evidence accumulation model, the Diffusion Model for Conflict (DMC) tasks (Ulrich et al., 2015). Similar to the central assumption of dual-route models, DMC assumes that a single decision-making process accumulates evidence to trigger a response by superimposing activations from automatic (distractor-based) and controlled (target-based) processes. However, the output of distractor-based processes follows a pulse-like function, meaning that the output of these processes first increases until a maximum and then decreases back to zero. As is illustrated in Figure 1, modeling suggests that this maximum could be reached quickly for distractor-based activation produced by the irrelevant stimulus location in the Simon task, whereas it could be reached relatively late for distractor-based activation produced by the irrelevant flankers in the Eriksen task. As a result, distractor-based activation might decay in the Simon task, but might increase in the Eriksen task, when being superimposed with activation produced by target processing. Now consider that responses are faster with speed compared to accuracy due to lowering the decision threshold: Whereas this should increase the Simon effect because distractor-based activation is higher for faster responses, this should reduce the Eriksen flanker effect because distractor-based activation is smaller for faster responses.

**Table 1:** Best-fitting parameters of the Diffusion Model for Conflict (DMC) tasks (Ulrich et al., 2015) to the experimental data of the four subconditions (i.e., Simon/Eriksen × Speed/Accuracy) and the corresponding derived peak latencies of the amplitude of the distractor-based process as well as weighted root-mean-square errors (RMSEs) averaged across participants

	Simon task		Eriksen task	
	Speed	Accuracy	Speed	Accuracy
*DMC best-fitting parameters*				
Amplitude *A* of distractor process	25.6 (1.5)	21.8 (1.6)	24.1 (1.3)	26.0 (1.5)
Scale (ms) *τ* of distractor process	82 (13)	112 (15)	114 (12)	164 (13)
Shape *a* of distractor process	1.74 (0.09)	1.63 (0.09)	2.11 (0.09)	1.90 (0.09)
Decision boundary *b*	47.7 (1.8)	72.3 (4.2)	44.6 (3.0)	72.2 (4.2)
Drift rate *μ*_c_ of target process	0.48 (0.03)	0.70 (0.03)	0.51 (0.04)	0.67 (0.03)
Mean residual time (ms) *μ*_R_	305 (7)	343 (5)	322 (7)	357 (8)
Variability residual time (ms) *σ*_R_	19 (3)	21 (3)	24 (4)	24 (3)
Shape *α*_s_ of starting point distribution	2.45 (0.11)	2.94 (0.12)	2.41 (0.10)	3.08 (0.14)
*DMC peak time (ms) of distractor process*				
*t*_peak_ = *τ ·* (*a −* 1)	45 (9)	57 (16)	113 (14)	128 (18)
Goodness-of-fit (*RMSE*)	39.60 (3.36)	22.36 (2.85)	39.17 (3.52)	22.09 (2.86)

Standard errors (SEs) of the means are given in parentheses

The fitting procedure used the R-package DEoptim as implemented within the R-package DMCfun (Mackenzie & Dudschig, under review). The step size was t = 1 ms and the diffusion constant was fixed at σ =4

As one way to investigate whether the specific time-course of distractor-based activation could explain the potential conflict task-dependent effects of SAT in our study on a mean RT level, we will combine a speed-accuracy manipulation with more fine-grained RT analyses at a distributional level. Instead of just computing means of the congruent and incongruent condition to calculate the mean congruency effect, it is also possible to plot the difference between the RT distributions of each condition to examine the size of the congruency effect as a function of (response) time (e.g., De Jong et al., 1994; Ridderinkhof, 2002b; Schwarz & Miller, 2012). The slope of these so-called delta plots could be seen as a direct marker of the time-course of distractor-based activation: For the Simon task, the resulting delta plots are primarily decreasing (e.g., Ellinghaus et al., 2017; Mittelstädt & Miller, 2020), whereas for the Eriksen task, the delta plots are primarily increasing (e.g., Burle et al., 2014; Ulrich et al., 2015). Observing such a distinct distributional pattern between Simon versus Eriksen tasks in the present SAT paradigm would suggest that the specific time-course of distractor-based activation needs to be considered when interpreting the effects of SAT manipulations on a mean RT level.

To better understand the SAT influences on Simon versus Eriksen flanker effects across time, we also fitted the observed behavioral data to the DMC model. As mentioned above, this model has been shown to plausibly account for both means and distributions of RTs in the two conflict tasks by specifying exactly how the time-course of distractor-based activation interacts with target processing (Ulrich et al., 2015). Hence, fitting the DMC model to the data obtained from the present Simon-Eriksen-SAT paradigm allows us to directly investigate whether this model can also reasonably account for decision-making with different sources of distracting information (i.e., irrelevant flankers vs. irrelevant location) under time pressure.^2^ As mentioned above, opposing SAT influences on congruency effects may already emerge by changes in decision boundaries if distractor-based activation reaches its peak generally earlier in the Simon than in the Eriksen task. As will be considered in the *Discussion,* another recently introduced conflict task model, the Activation Suppression Race Model (ASR; Miller & Schwarz, in press), also assumes time-based suppression of distractor-based processes, and we consequently also explored whether and how this model can account for the empirical results. Overall, these model-based analyses help us to identify the processes within each task that modulate task performance based on the SAT manipulation.

## Method

### Participants

Thirty-two people (25 women) were tested at the University of Tübingen. They ranged in age from 18 to 36 years (M = 23.6) and 25 were right-handed. All participants gave informed consent before testing and they were tested in a single session lasting approximately 45 min. Participants received either course credits or money (10€) for participation.^3^

### Apparatus and stimuli

Stimulus presentation and recording of responses were controlled by E-Prime 2. All visual stimuli were presented in a white font on a black background of a monitor running with a refresh rate of 60 Hz and were viewed from a distance of approximately 60 cm. A centrally positioned white plus sign (+) served as the fixation point. The stimuli were letters (i.e., H and S) that subtended approximately 1*.*4° with a monospaced font. For each participant, the two stimulus letters were randomly assigned to left- and right-hand responses. In Simon task blocks, the target letter appeared approximately 5*.*3^*o*^ to the left or right of the center of the screen (measured to the center of the letter). In the Eriksen task blocks, the target letter was centrally presented and two flanker letters appeared on each side of the target letter (e.g., HHSHH), with a separation of approximately 0*.*1° between letters. Responses were key presses with the left and right index fingers on the “Y” and “-” keys of a QWERTZ computer keyboard.

### Procedure

Task (Simon vs. Eriksen) and speed-accuracy condition (speed high vs. accuracy high, hereafter SP and AC) were held constant within a block and varied in a predictable order across blocks. Specifically, participants repeatedly performed loops of each of the four possible block types over the course of the experiment (e.g., block 1: Simon-SP, block 2: Eriksen-SP, block 3: Simon-AC, block 4: Eriksen-AC, block 5: Simon-SP, block 6: Eriksen-SP...) with task order and speed-accuracy condition order counterbalanced across participants (i.e., in total four different counterbalanced orders). Half of the participants were tested with four-block loops in which the Simon task was always presented in the first and third blocks and the Eriksen task in the second and fourth blocks. Furthermore, half of the participants were tested in the SP condition for the first and second blocks and in the AC condition for the third and fourth blocks. The remaining participants received the reverse order of task and speed-accuracy condition, respectively. Thus, all participants always performed two successive blocks of one speed-accuracy condition and tasks alternated blockwise. Each of the 28 blocks consisted of 32 randomly ordered trials, with eight presentations of each of the four possible stimulus displays in the Eriksen task (i.e., two possible target letters *×* two flanker letters) and in the Simon task (i.e., two possible target letters *×* two locations). Instructional screens at the beginning of each block served as a reminder of the stimulus-response mapping, upcoming task, and speed-accuracy requirements. After the first four practice blocks, the experimenter further encouraged participants to follow the SP and AC instruction.

For SP blocks, participants were instructed to emphasize response speed and be less concerned about making errors, and they only received feedback about their mean RT after performing SP blocks. In order to avoid a complete guessing strategy, participants received an additional message after SP blocks if there were more than ten response errors within a block (“You made many errors – you should be fast but without guessing”). For AC blocks, participants were instructed to concentrate on making accurate responses without losing too much speed, and they only received feedback about their number of correct trials after performing AC blocks.

At the beginning of each trial, the fixation cross appeared on the screen for 500 ms. Following the offset of the fixation cross, a single letter was presented to the left or right side of the screen (i.e., Simon task) or the letter array was presented on the center of the screen (i.e., Eriksen task). The stimulus or stimuli remained on the screen until participants responded. After each response, feedback indicated whether the response was (1) “correct!” (2) “error,” (3) “too slow!” or (4) “too fast” (if RT < 100 ms). Each block of trials used a fixed RT deadline to calculate “too slow!” feedback, which depended on the specific speed-accuracy block type condition. For Simon-SP and Eriksen-SP blocks, the RT deadlines were set to 500 ms (750 ms in practice blocks). For Simon-AC and Eriksen-AC blocks, the RT deadlines were set to 2.5 s (2.75 s in practice blocks). RT deadlines were based on intensive pre-testing and the results of the pilot experiment (Appendix A) showed that the specific RT deadlines were appropriate. Feedback was displayed in white or red font for either 1 s or for 2.5 s depending on speed-accuracy requirements. Specifically, in SP blocks, to further encourage fast responses, feedback was always displayed in white font for 1 s except when the RT deadline was not met, in which case it was displayed in red font for 2.5 s. In AC blocks, to further encourage accurate responses, feedback was always displayed in white font for 1 s except when a response error was made, in which case it was displayed in red font for 2.5 s.

## Behavioral results

The first four practice blocks were excluded from any analyses. For RT analyses, we excluded choice error trials (10.2%).

### Reaction times (RTs)

**Fig. 2:**
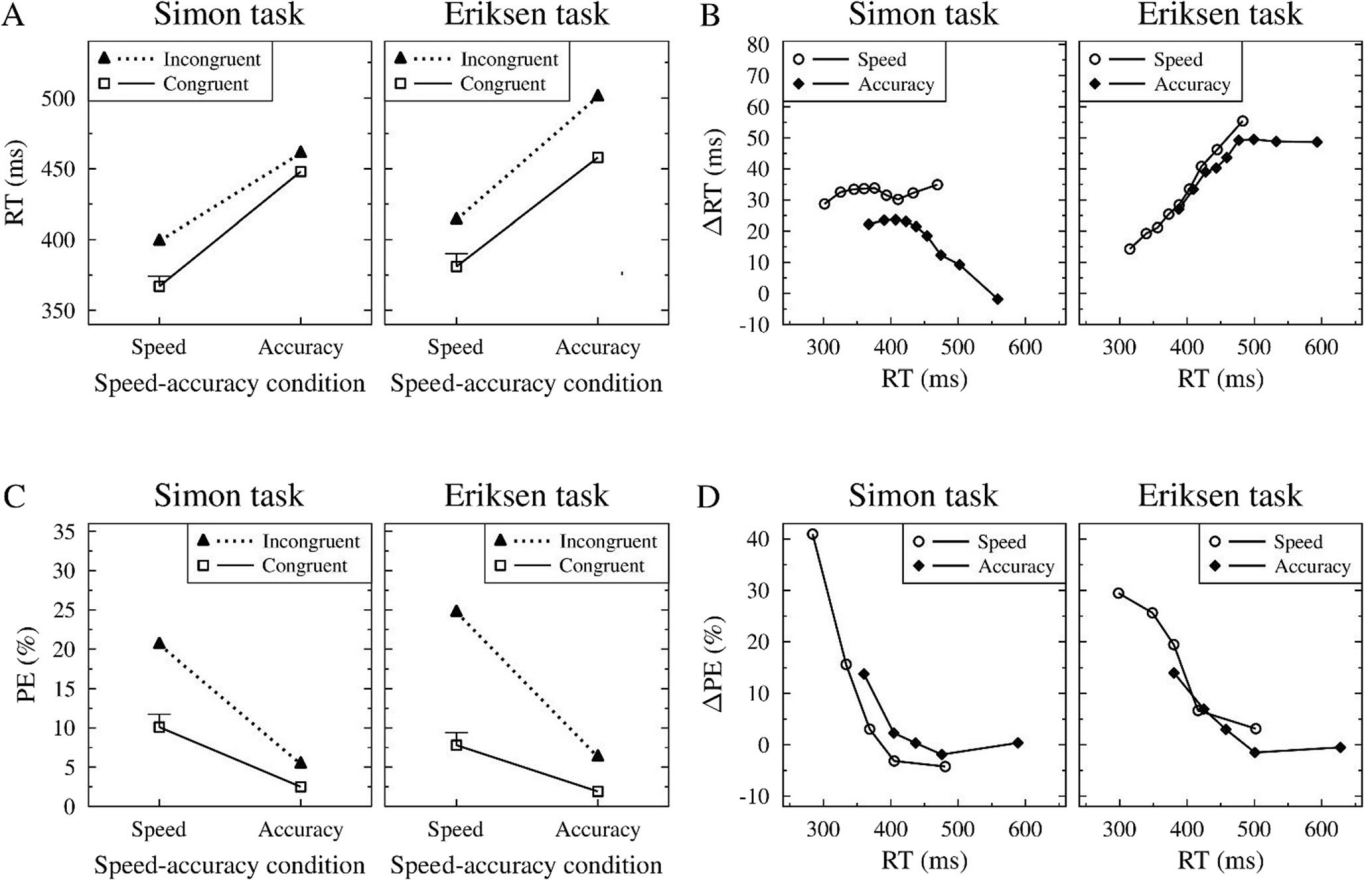
(**A**) Mean reaction time (RT) as a function of speed-accuracy condition (speed, accuracy) and congruency (congruent, incongruent) separately for the Simon and Eriksen tasks. (**B**) Delta plots showing incongruent minus congruent differences in mean RT within each of 10 RT percentiles, plotted against the quantile average RTs, separately for each speed-accuracy condition (speed, accuracy) *×* task condition (Simon, Eriksen). (**C**) Mean percentage error (PE) as a function of speed-accuracy condition and congruency separately for the Simon and Eriksen tasks. (**D**) Delta plots showing incongruent minus congruent differences in mean PE within each of 5 RT quantiles, plotted against the quantile mean RTs, separately for each combination of speed-accuracy condition (speed, accuracy) and task (Simon, Eriksen). The error bars in A and C indicate 1 *SE* (standard error) based on the pooled error terms of two main effects and the interaction in the specific conflict task (i.e., Simon vs. Eriksen task)

Figure 2A shows the mean RTs as a function of speed-accuracy condition (SP, AC) and congruency (congruent, incongruent) separately for the Simon and Eriksen task.

A repeated-measures ANOVA with the within-subject factors of speed-accuracy condition, congruency, and task revealed significant main effects of speed-accuracy condition, *F*(1, 31) = 77.89, *p < .*001, *η*_*p*_^2^ = .72, congruency, *F*(1, 31) = 167.3, *p < .*001, *η*_*p*_^2^ = .84 and task, *F*(1, 31) = 40.15, *p < .*001, *η*_*p*_^2^ = .56. The mean RT was smaller in SP than in AC blocks (390 ms vs. 467 ms), the mean RT was smaller in congruent than in incongruent trials (413 ms vs. 444 ms) and there was also a smaller mean RT in the Simon than in the Eriksen task (419 ms vs. 438 ms). Two two-way interactions were significant: Specifically, the interaction between congruency and task, *F*(1, 31) = 24.32, *p < .*001, *η*_*p*_^2^ = .44, indicated that the Simon effect (430 – 408 = 22 ms) was smaller than the flanker effect (457 – 419 = 38 ms). The interaction between speed-accuracy condition and task, *F*(1, 31) = 5.90, *p* = *.*021, *η*_*p*_^2^ = .16, indicated that the RT difference between SP and AC blocks was more pronounced for the Eriksen (479 – 397 = 82 ms) than for the Simon task (455 – 383 = 72 ms). Most important with regard to our preregistered hypothesis, there was also a significant three-way interaction between all three factors, *F*(1, 31) = 26.65, *p < .*001, *η*_*p*_^2^ = .46. As expected, the Simon effect was larger in SP compared to AC blocks (32 ms vs. 13 ms), whereas the flanker effect was smaller in SP compared to AC blocks (32 ms vs. 43 ms). Separate ANOVAs for each conflict task with the factors speed-accuracy condition and congruency yielded significant interactions for both the Simon task, *F*(1, 31) = 16.30, *p < .*001, *η*_*p*_^2^ = .34, and the Eriksen task, *F*(1, 31) = 4.32, *p* = *.*046, *η*_*p*_^2^ = .12.

Next, we constructed delta plots to examine the time-courses of the two congruency effects separately for SP and AC blocks. Specifically, we created RT percentiles (10%, 20%, 30%, ..., 90%) separately for each participant within each of eight conditions (i.e., SP/AC *×* congruent/incongruent *×* Simon/Eriksen).^4^ As can be seen in Fig. 2B, the delta plots for the Simon and Eriksen flanker tasks followed qualitatively distinct time-courses. Specifically, the flanker delta plots generally increased across the whole RT distribution, whereas the Simon delta plots showed the typical decreasing time-course for larger RTs (i.e., > 430 ms) in AC blocks, but a relatively stable time-course across the whole RT distribution in SP blocks. To compare the time-courses of the delta plots, we summarized the delta plot for each participant and each condition with a linear regression model predicting the delta in each bin from the mean RT in that bin. An ANOVA with factors of task and speed-accuracy condition on the mean slopes only revealed that the two main effects were significant (with *p > .*934 and *η*_*p*_^2^ < .01 for the interaction). The main effect of task reflected larger slopes for the Eriksen (0.19) than for the Simon task (-0.06), *F*(1, 31) = 64.63, *p < .*001, *η*_*p*_^2^ = .68. The main effect of speed-accuracy condition reflected larger slopes in SP (0.14) compared to AC blocks (0.00), *F*(1, 31) = 10.77, *p* = *.*003, *η*_*p*_^2^ = .26.

### Percentage errors (PEs)

Figure 2C shows the mean PEs in the corresponding conditions. An ANOVA parallel to the one conducted on mean RT yielded significant main effects of speed-accuracy condition, *F*(1, 31) = 65.33, *p < .*001, *η*_*p*_^2^ = .68, and congruency, *F*(1, 31) = 134.77, *p < .*001, *η*_*p*_^2^ = .81. Error rates were lower in AC than in SP blocks (4.0% vs. 16.4%) and error rates were also lower in congruent than in incongruent trials (5.9% vs. 14.6%). Furthermore, the two-way interaction between speed-accuracy condition and congruency was significant, *F*(1, 31) = 69.22, *p < .*001, *η*_*p*_^2^ = .69, reflecting larger congruency effects in SP (23.3% – 9.6% = 13.7%) compared to AC blocks (5.9% – 2.2% = 3.7%). In addition, there was a significant two-way interaction between congruency and task *F*(1, 31) = 5.67, *p* = *.*024, *η*_*p*_^2^ = .16, indicating that Simon effects were generally smaller (13.2% – 6.5% = 6.7%) than flanker effects (15.9% – 5.2% = 10.7%). Finally, there was a significant three-way interaction between all three-factors, *F*(1, 31) = 4.84, *p* = *.*035, *η*_*p*_^2^ = .14, indicating that the increase of congruency effects in SP compared to AC blocks was more pronounced for the Eriksen (4.4% vs. 17.0%) than for the Simon task (2.9% vs. 10.5%). No other effects were significant (with all *p*s *> .*195 and all *η*_*p*_^2^s *< .*054).

For completeness, we also constructed delta plots for the error rates. For each participant and condition (speed-accuracy condition, congruency, task), we rank-ordered the individual RTs (including both correct responses and errors) and then computed the error rate within each bin. Figure 2D shows the mean PEs plotted against the mean RT bins separately for each task and speed-accuracy condition. As can be seen in this figure, the congruency effects in error rates continually decreased with slower responses for all conditions.

## Diffusion Model for Conflict (DMC) tasks modeling

The DMC model assumes that the outputs of controlled (target-based activation) and automatic (distractor-based activation) processes are superimposed into a single Wiener diffusion process (with the diffusion constant *σ*) toward the correct decision boundary *b*. The drift rate of this process is determined at each time point *t* by the combined inputs from the temporally constant input of a target-based process with drift rate *μ*_c_ and the time-varying input of a distractor-based process with drift rate *μ*_i_(*t*). Specifically, the input from the distractor-based process is modeled as a pulse-like gamma density function with shape parameter *a* which reaches its peak amplitude *A* at time *t*_peak_ = (*a −* 1) *· τ*, after which it decreases back to zero. RT in a given trial is the sum of the decision time needed to reach the response boundary *b* plus a normally distributed non-decision (residual) time (i.e., with *μ*_R_ and *σ*_R_). Starting point variability is implemented by sampling from a beta-shaped distribution *B*, which varies symmetrically around zero from *b*_1_ to *b*_2_.

The DMC model was fit to the observed individual data of the four experimental conditions (i.e., Simon/Eriksen *×* Speed/Accuracy) from each participant by using the R-package DMCfun (Mackenzie & Dudschig, 2021). Specifically, as is elaborated in more detail in Appendix B, the model was fitted simultaneously to the individual and condition-specific errors and RT distributions by minimizing the root-mean-squared error (RMSE) between observed and predicted values.

### Fitting results

**Fig. 3:**
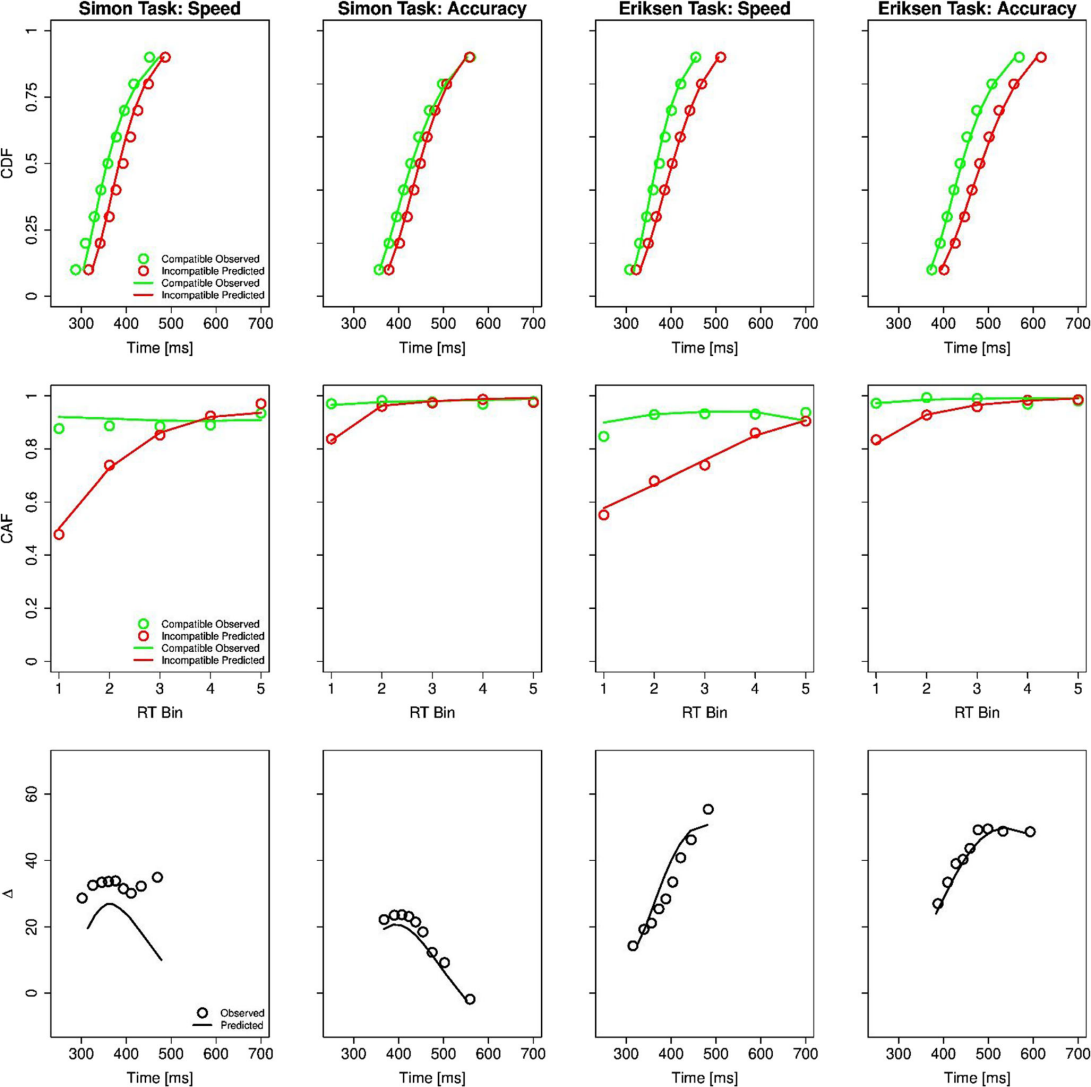
Experimental results and predictions of the Diffusion Model for Conflict (DMC) tasks. The panels within each column depict the fitting results of one the four subconditions (i.e., Simon/Eriksen *×* Speed/Accuracy). The panels within each row depict cumulative distribution function (CDF) of correct reaction times (RTs) separately for congruent and incongruent trials, conditional accuracy functions (CAF) separately for congruent and incongruent trials, RT delta plots showing incongruent minus congruent differences in mean RTs within each of 10 deciles plotted against the decile averages, respectively

**Figure B1.**
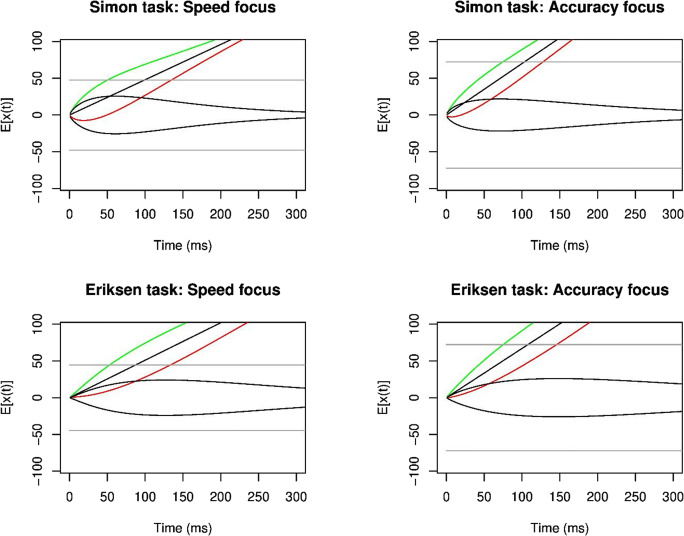
Mean activation functions based on simulation results with the best-fitting parameters displayed in Table 1 of the Diffusion Model for Conflict tasks (Ulrich et al., 2015). For each condition, 100,000 trials with a stepsize of t = 1 ms were simulated. Solid black lines depict target-based activations, dotted colored lines depict congruent (green) and incongruent (red) distractor-based activations, solid colored lines depict the superimposed activations in the congruent (green) and incongruent (red) condition, grey lines depict upper and lower decision boundaries, respectively

The mean best-fitting parameters and mean RMSEs as a function of task and speed-accuracy condition are shown in Table 1, and the corresponding model fits to capture the distributional RT and error data are visualized in Fig. 3. In addition, the activation functions based on the best-fitting parameters are visualized in Fig. B1 in Appendix B. As can be seen in Fig. 3, the DMC model provides a reasonable fit to the data. In the following, we briefly elaborate how the corresponding parameters were modulated by our experimental manipulations while reporting the results of repeated-measures ANOVAs with the two factors task (Eriksen, Simon) and speed-accuracy condition (SP, AC) on the estimated parameter values and on the derived time-course of distractor-based activation.

As mentioned above, distractor-based activation peaks at time *t*_peak_ = (*a −* 1) *· τ* and a significant main effect of task indicated that this peak was reached earlier, on average, in the Simon task (*t*_peak_ = 51 ms) than in the Eriksen task (*t*_peak_ = 121 ms), *F*(1, 31) = 63.56, *p < .*001, *η*_*p*_^2^ = .67 (with all other *p*s *> .*473 and *η*_*p*_^2^s *< .*02). Thus, the time-course of distractor-based processes was mainly modulated by task, suggesting faster location-based than flanker-(letter-)based processing.^5^ Interestingly, with regard to the strength of distractor-based processes *A*, there was a significant interaction, *F*(1, 31) = 5.64, *p* = *.*024, *η*_*p*_^2^ = .15 (with all other *p*s *> .*431 and *η*_*p*_^2^s *< .*02). As can be seen in Table 1, the amplitude of distractor-based activation was slightly larger in SP than in AC in the Simon task, but slightly larger in AC than in SP in the Eriksen task. Thus, in addition to task-specific differences in the speed of distractor-based processes, suppression of distractor-based activation seems to operate less (more) efficiently under time pressure in the Simon (Eriksen) task.

As expected, decision boundaries were on average smaller with SP (*b* = 46.2) compared to AC focus (*b* = 72.3) as indicated by a significant main effect of speed-accuracy condition, *F*(1, 31) = 40.11, *p < .*001, *η*_*p*_^2^ = .56 (with all other *p*s *> .*398 and *η*_*p*_^2^s *< .*03). The ANOVA on drift rates *μ*_c_ of the target-based process also revealed a significant main effect of speed-accuracy condition, *F*(1, 31) = 30.35, *p < .*001, *η*_*p*_^2^ = .50. In line with previous findings (e.g., Dambacher & Hübner, 2015; Servant et al., 2019), there was less efficient evidence accumulation with speed (*μ*_c_ = 0.50) compared to accuracy focus (*μ*_c_ = 0.69). Interestingly, this effect was more pronounced in the Simon task than in the Eriksen task, as reflected in a marginally significant interaction, *F*(1, 31) = 4.10, *p* = *.*051, *η*_*p*_^2^ = .12 (with *p* = *.*812 and *η*_*p*_^2^ *< .*01 for the main effect of task). The ANOVA on mean residual times *μ*_R_ yielded significant main effects of task, *F*(1, 31) = 14.83, *p* = *.*001, *η*_*p*_^2^ = .32, and speed-accuracy condition, *F*(1, 31) = 21.69, *p < .*001, *η*_*p*_^2^ = .41, with no interaction (*p* = *.*626, *η*_*p*_^2^ < .01). Residual times were on average smaller in the Simon task (*μ*_R_ = 324 ms) than in the Eriksen task (*μ*_R_ = 339 ms) and also smaller with SP (*μ*_R_ = 313 ms) compared to AC (*μ*_R_ = 350 ms) focus. Furthermore, there was no evidence that the variability of residual times *σ*_R_ differed across conditions (all *p*s *> .*140 and all *η*_*p*_^2^s *< .*07). Finally, the shape parameter *α*_s_ of the starting point distribution was smaller with speed (*α*_s_ = 2.43) compared to accuracy (*α*_s_ = 3.01) as reflected in a significant main effect of speed-accuracy, *F*(1, 31) = 25.37, *p < .*001, *η*_*p*_^2^ = .45 (all other *p*s *> .*269 and all *η*_*p*_^2^s *< .*04). This finding suggests that there was more trial-to-trial variability in the starting point of the diffusion process under time pressure.

In sum, the fitting results demonstrate substantial between-task differences regarding the time-course of distractor-based activation and its suppression as a function of time pressure. Furthermore, there were multiple effects of time pressure on processing (i.e., decision boundaries, rate of evidence accumulation, non-decision times), but the latter effects were generally consistent across tasks. Thus, the fitting results reinforce the idea that an interaction of decision boundaries and the task-specific time-course of distractor-based activation is sufficient to explain the observed opposing effects of SAT on task performance (i.e., RTs) across these two tasks. Of course, a subtle combination of other processes may also contribute to the interactive effects on behavior, but it seems difficult to see how the essentially similar directional effects of the SAT manipulation on multiple processes alone could produce the counteracting (conflict-task specific) effects on task performance without also assuming differential time-courses of distractor-based activation.

## Discussion

The present study demonstrates that time pressure can have counteracting effects on task performance in decision-making under conflict. Specifically, using a Simon-Eriksen-SAT paradigm, we showed that the mean congruency effect on RT increased in the Simon task but decreased in the Eriksen task when the focus was on response speed versus response accuracy. The observed between-task differences of congruency effects on a distributional RT level (i.e., primarily decreasing or stable delta plots in the Simon task but primarily increasing delta plots in the Eriksen task) suggest that distractor-based activation unfolded differently in time across the two conflict tasks. Modeling further reinforced the idea that this result pattern can be reconciled within the architecture of evidence accumulation models by assuming that the time-course of distractor processing can produce opposing effects on target processing under varying decision boundaries.

In general, growing evidence suggests that time pressure affects processing at several levels (e.g., Steinemann et al., 2018). For example, the effects of SAT manipulations in the Eriksen and Simon conflict tasks can be attributed to several parameters within evidence accumulation including changes in the height of decision boundaries (e.g., Hedge et al., 2019), the drift rate of perceptual evidence accumulation (e.g., Dambacher & Hübner, 2015), and non-decision (motor) times (e.g., Rinkenauer et al., 2004; Van der Lubbe et al., 2001). In line with these previous findings, the current DMC model-fitting results revealed within a single study that drift rates, non-decision times, and decision boundaries decreased with increased time pressure when making decisions under different sources of potentially conflicting information (i.e., location and flankers). More importantly, these modeling results revealed differences in the time-course of distractor-based activation between tasks – as also becomes evident from the task-specific delta functions. This means that the empirical and modeling data are generally consistent with the predictions of a time-varying distractor-based activation account like the one illustrated in Fig. 1 (see also Fig. B1 in Appendix B). Following Occam’s razor, we thus suggest that the standard SAT account of varying decision boundaries can account for the present results just by assuming that the temporal overlap of distractor- and target-based activations differs across the two conflict tasks. Thus, our results are well in line with recent suggestions that the inputs from time-varying (initially increasing but then decreasing) distractor-based processes superimpose with the inputs from target-based processes to drive evidence accumulation towards the correct decision boundary in conflict tasks (e.g., Hübner & Töbel, 2019; Luo & Proctor, 2020; Miller & Schwarz, in press; Ulrich et al., 2015).

Although we have conceptualized the present study within the DMC model, other quantitative conflict task models are also capable of producing both positive- and negative-going delta plots (e.g., Hübner & Töbel, 2019; Miller & Schwarz, in press; Schwarz & Miller, 2012; Wühr & Heuer, 2018), and hence these models may well also account for the empirical result pattern. For example, Miller and Schwarz (in press) recently introduced the activation suppression race (ASR) model, which assumes a race between suppression of the distractor-based activation produced from irrelevant information (process A) and recognition of the relevant information (process B) before decision-making and motor processes take place (process C). The durations of processes A and B were assumed to be exponentially distributed with means of *τ*_*A*_ and *τ*_*B*_, respectively, whereas the duration of process C was assumed to be normally distributed with mean and standard deviation *μ*_*C*_ and *σ*_*C*_. When suppression is not completed before central processing begins (i.e., duration of A > duration of B), distractor-based activation produces harmful interference in incongruent trials – that is, it increases the duration of the subsequent process C by an increment *λ*_inh_ representing response inhibition. Initial examination revealed that the ASR model can reasonably capture the empirical RT pattern with changes in parameter values that seem generally well in line with the present conclusions (and DMC model fitting results). Specifically, the time needed for pre-decision, decision, and motor processes decreased under time pressure for both of the conflict tasks (i.e., *τ*_B,SP_ *< τ*_B,AC_ and *μ*_C,SP_ *< μ*_C,AC_), and the average time needed for suppressing distractor-based activation was less in the Eriksen task than in the Simon task (i.e., *τ*_A,Eriksen_ *< τ*_A,Simon_). The net result was that the probability of interference was larger with speed compared to accuracy focus in the Simon task, whereas the reverse was true in the Eriksen task. This shows that task-specific distractor-based processes can also produce counteracting effects under time pressure within the ASR processing architecture (see Appendix C for more details about the fits of the ASR model).

The specific causes underlying the timing of distractor-based processing could be further elucidated. For example, the decreasing impact of distractor-based activation with slower responses (as evident in the Simon task) is in line with non-quantiative accounts assuming passive decay (e.g., Hommel, 1993, 1994) and/or active inhibition (e.g., Ridderinkhof, 2002a, 2002b) of distractor-based activation. The time-based processing models (DMC and ASR) discussed here might be considered as quantitative versions of these earlier accounts, and the present empirical and fitting results are in principle consistent with both passive decay and active suppression of distractor-based activation. Importantly, however, the quantitative models provide a precise and parsimonious mechanistic explanation of why the two conflict effects should differentially change under time pressure. Because location-based information in the Simon task is processed faster than flanker-based information in the Eriksen task, there is a greater temporal overlap from distractor- to target-based activation in the Simon than in the Eriksen task. Thus, the distractor-based activation in the Simon (but not in the Eriksen task) is already fading out under both speed and accuracy focus when being superimposed with target-based activation, but passive decay and/or active suppression has taken place to a smaller degree under time pressure. In other words, the important aspect seems to be the *relative* speed of distractor versus target processing.

Therefore, the present findings do not demonstrate that time-based processes alone are sufficient to explain all aspects of conflict processing. Thus, the opposing effects of time pressure in the Eriksen and Simon tasks may also be, at least partially, the result of task-specific processing adjustments – in particular, because conflict resolution in these tasks likely involves partially distinct control mechanisms (e.g., Egner, 2007). For example, in the Eriksen task, attentional control processes primarily modulate perceptual and decision-making processes when dealing with conflict arising from a mismatch between target and flanker identities (e.g., Lavie & Tsal, 1994; Miller, 1991; White et al., 2011). In the Simon task, however, control processes primarily act on a motor level because conflict emerges from a mismatch between target location and response location (Hasbroucq et al., 1999; Mittelstädt & Miller, 2018; Servant et al., 2016. In other words, although incorrect motor activation is observed in both Simon (e.g., Stürmer et al., 2002; Van der Lubbe et al., 2001) and Eriksen tasks (e.g., Gratton et al., 1988; Mattler, 2003; Servant et al., 2015), motor-specific conflict resolution processes might play a particularly important role in the Simon task (e.g., Stürmer & Leuthold, 2003). Because time pressure speeds up late motoric processes in addition to lowering decision boundaries, chances are smaller that incorrect motor activation can be corrected in the Simon task, and this would be reflected in relatively large Simon effects (cf. Burle et al., 2014; Schlaghecken & Eimer, 2002). Interestingly, some preliminary support for the idea of such task-specific modulations of motor processes comes from both the fitting and empirical results. Specifically, there was evidence for a greater amplitude increase in distractor-based activation under time pressure in the Simon task than in the Eriksen task for the fitting results of both the DMC and ASR model. Furthermore, the SAT manipulation appears to shift the delta plots up and down in the Simon task (but not in the Eriksen task) as has been observed with other motor manipulations (Mittelstädt & Miller, 2020).

However, the results suggest that time-dependent processes need to be considered before more control-based attentional mechanisms can be inferred (e.g., Hawkins & Heathcote, 2021; Hübner & Töbel, 2019; Logan, 1980; Mittelstädt & Miller, 2020; Weichart et al., 2020). Critically, although the present study highlighted this issue by illuminating the effect of time pressure on congruency effects for standard versions of visual Simon and Eriksen flanker tasks, time-varying distractor-based processes seem to influence behavior in many other versions of conflict tasks. For example, primarily decreasing delta plots have also been observed for auditory Simon effects (Xiong & Proctor, 2016) and priming effects (Ellinghaus & Miller, 2018; Schlaghecken et al., 2011), and, conversely, primarily increasing delta plots have also been observed for tactile flanker effects (Baciero et al., 2021), SNARC effects (Moro et al., 2018), and manual Stroop effects (Kinoshita et al., 2017; Pratte et al., 2010). Consistent with the present conclusions, for example, speed instructions reduced the size of manual Stroop effects, and these instructions profoundly affected the decision bounds with narrower bounds under speed than accuracy instructions (Hedge et al., 2019). Furthermore, the temporal dynamics of activations related to different distractor types may also critically affect modulations of conflict effects across different tasks (i.e., congruency sequence effects; cf. Schlaghecken & Maylor, 2020).

On a broader level, the present results also provide further support for the idea of moving beyond mean RT when examining the effects of experimental manipulations in non-conflict tasks (Heathcote et al., 1991; Schweickert & Giorgini, 1999; Van Zandt, 2002), because time-based processing mechanisms seem to contribute to performance in many different contexts, such as task switching (e.g., Altmann & Gray, 2002; Mittelstädt et al., 2019), multitasking (e.g., Fischer et al., 2018; Miller et al., 2009), mental rotation (e.g., Liesefeld et al., 2015), emotional processing (e.g., Sharma & McKenna, 2001; Yap & Seow, 2014; Zhou et al., 2016), and visual search (e.g., Müller & Rabbitt, 1989; Schwarz & Miller, 2016).

## Figures and Tables

**Figure A1.**
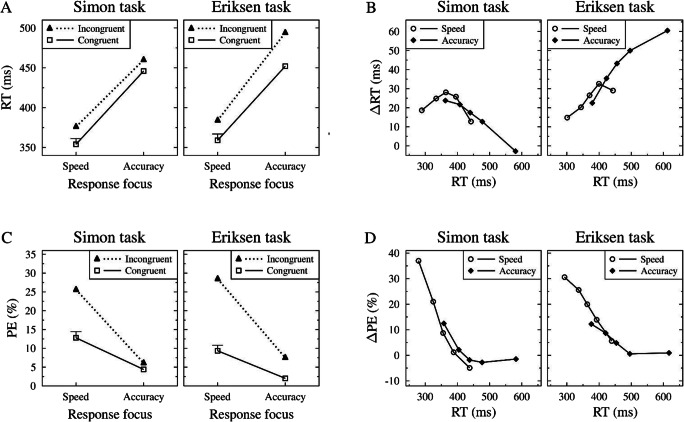
(**A**) Mean reaction time (RT) as a function of speed-accuracy condition (speed, accuracy) and congruency (congruent, incongruent) separately for the Simon and Eriksen tasks. (**B**) Delta plots showing incongruent minus congruent differences in mean RT within each of five RT quantiles, plotted against the quantile average RTs, separately for each speed-accuracy condition (speed, accuracy) *×* task (Simon, Eriksen) condition. (**C**) Mean percentage error (PE) as a function of speed-accuracy condition and congruency separately for the Simon and Eriksen tasks. (**D**) Delta plots showing incongruent minus congruent differences in mean PE within each of five RT quantiles, plotted against the quantile mean RTs, separately for each combination of speed-accuracy condition (speed, accuracy) and task (Simon, Eriksen). The error bars in A and C indicate 1 *SE* (standard error) based on the pooled error terms of two main effects and the interaction in the specific conflict task (i.e., Simon vs. Eriksen task)

**Figure C1.**
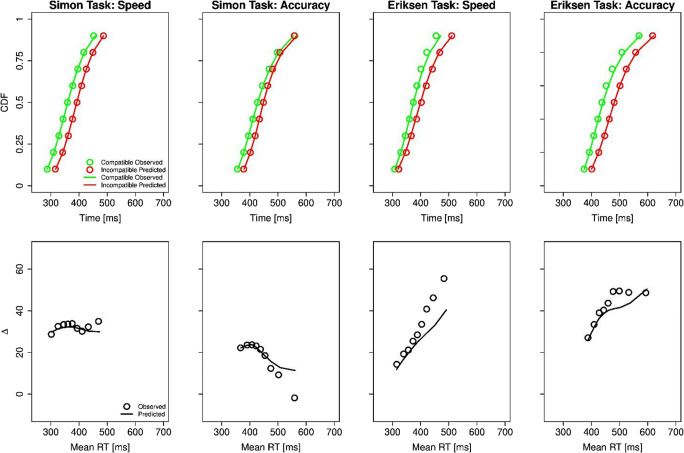
Experimental results and predictions of the Activation Suppression Race (ASR) model. The panels within each column depict the fitting results of one the four subconditions (i.e., Simon/Eriksen *×* Speed/Accuracy). The panels within each row depict cumulative distribution function (CDF) of correct reaction times (RTs) separately for congruent and incongruent trials, or RT delta plots showing incongruent minus congruent differences in mean RTs within each of 10 deciles plotted against the decile averages, respectively

## Appendix A

### Pilot experiment

In this appendix, we describe the results of our pilot experiment. This experiment was similar to the main experiment except that stimuli were only presented until the RT deadline and no response was recorded when participants did not respond during stimulus presentation (i.e., “too slow”). We made this procedural choice on purpose in the hope of increasing the effect of the SAT manipulation, but we overlooked the fact that this artificially truncates the RT distribution (we are grateful to Mathieu Servant for making us aware about this issue). On the one hand, one might argue that this is not a problematic issue, because our main conclusions concern differences between Simon and Eriksen tasks and this truncation would have probably affected both tasks in the same way (and the percentage of “too-slow” responses was small, see Results section.) On the other hand, however, we cannot entirely rule out that this truncation might have potentially contaminated our central (mean) RT finding. To directly address this concern, we decided to conduct the experiment reported in the main text without “no response” trials to investigate whether an SAT manipulation has opposing SAT effects on the two mean congruency RTs. The untruncated RT data obtained from the main experiment also allowed us to fit the data to the DMC model. For the sake of transparency, we decided to report this experiment including no response trials as a pilot experiment in this appendix.

### Method

***Participants.*** Forty people (34 women) were tested at the University of Tübingen.^6^ They ranged in age from 19 to 35 years (M = 23.8) and 35 were right-handed. All participants gave informed consent before testing and they were tested in a single session lasting approximately 45 min. Participants received either course credits or money (10€) for participation.

***Apparatus, stimuli, and procedure.*** The apparatus, stimuli, and procedure were the same as for the experiment described in the main text except as we used an explicit (not an implicit RT deadline). More precisely, the stimulus or stimuli remained on the screen until participants responded, up to a maximum which depended on the specific block type (see main text). Thus, no response could be given in “too-slow” trials.

### Results

The first four practice blocks were excluded from any analyses. For both percentage error (PE) and RT analyses, we excluded “too-fast“ (0.1%) and “too-slow“ (3.5%) trials. For the SP condition, we excluded 5.3% congruent and 6.8% incongruent trials in Simon blocks and 5.2% congruent and 10.3% incongruent in Eriksen blocks. For the AC condition, we excluded 0.1% congruent and <0.1% incongruent trials in Simon blocks and 0.3% congruent and 0.3% incongruent in Eriksen blocks. For RT analyses, we additionally excluded choice error trials (11.6%).

***Reaction times (RTs).*** Figure A1A shows the mean RTs as a function of speed-accuracy condition (SP, AC) and congruency (congruent, incongruent) separately for the Simon and Eriksen task.

A repeated-measures ANOVA with the within-subject factors of speed-accuracy condition, congruency, and task revealed significant main effects of speed-accuracy condition, *F*(1, 39) = 125.62, *p < .*001, *η*_*p*_^2^ = .76, congruency, *F*(1, 39) = 323.99, *p < .*001, *η*_*p*_^2^ = .89 and task, *F*(1, 39) = 33.87, *p < .*001, *η*_*p*_^2^ = .46. The mean RT was smaller in SP than in AC blocks (368 ms vs. 463 ms), the mean RT was smaller in congruent than in incongruent trials (402 ms vs. 428 ms) and there was also a smaller mean RT in the Simon than in the Eriksen task (409 ms vs. 422 ms). All two-way interactions were significant: Specifically, there was a significant two-way interaction between congruency and task, *F*(1, 39) = 31.74, *p < .*001, *η*_*p*_^2^ = .45, indicating that the Simon effect (418 – 400 = 18 ms) was smaller than the flanker effect (439 – 405 = 34 ms). The significant two-way interaction between speed-accuracy condition and task, *F*(1, 39) = 6.91, *p* = *.*012, *η*_*p*_^2^ = .15, indicated that the RT difference between SP and AC blocks was more pronounced for the Eriksen (473 – 371 = 102 ms) than for the Simon task (453 – 365 = 88 ms), and the significant two-way interaction between speed-accuracy condition and task, *F*(1, 39) = 5.50, *p* = *.*024, *η*_*p*_^2^ = .12, reflected slightly smaller average congruency effects in SP (380 – 356 = 24 ms) than in AC blocks (477 – 449 = 28 ms). Most important with regard to our preregistered hypothesis, there was also a significant three-way interaction between all three factors, *F*(1, 39) = 119.77, *p < .*001, *η*_*p*_^2^ = .41. As expected, the Simon effect was larger in SP compared to AC blocks (22 ms vs. 14 ms), whereas the flanker effect was smaller in SP compared to AC blocks (25 ms vs. 42 ms). Separate ANOVAs for each conflict task with the factors speed-accuracy condition and congruency yielded significant interactions for both the Simon task, *F*(1, 39) = 5.23, *p* = *.*028, *η*_*p*_^2^ = .12, and the Eriksen task, *F*(1, 39) = 31.68, *p < .*001, *η*_*p*_^2^ = .45.

Next, we constructed delta plots to examine the time-courses of the two conflict effects separately for SP and AC blocks. Specifically, we rank-ordered each participants’ RTs within each of eight conditions (i.e., SP/AC *×* congruent/incongruent *×* Simon/Eriksen), and then partitioned the RTs of each condition into five bins.^7^ As can be seen in Fig. A1B, the delta plots for the Simon and Eriksen flanker tasks followed qualitatively distinct time-courses. Specifically, the flanker delta plots generally increased across the whole RT distribution, whereas the Simon delta plots showed the typical decreasing time-course for larger RTs (i.e., > 360 ms).

***Percentage errors (PEs).*** Figure A1C shows the mean PEs in the corresponding conditions. An ANOVA parallel to the one conducted on mean RT yielded significant main effects of speed-accuracy condition, *F*(1, 39) = 131.88, *p < .*001, *η*_*p*_^2^ = .77, and congruency, *F*(1, 39) = 153.46, *p < .*001, *η*_*p*_^2^ = .80. Error rates were lower in AC than in SP blocks (5.0% vs. 19.0%) and error rates were also lower in congruent than incongruent trials (7.1% vs. 16.9%). Furthermore, the two-way interaction between speed-accuracy condition and congruency was significant, *F*(1, 39) = 116.27, *p < .*001, *η*_*p*_^2^ = .75, reflecting larger congruency effects in SP (27.0% – 11.1% = 15.9%) compared to AC blocks (6.8% – 3.2% = 3.6%). Finally, there was a significant two-way interaction between congruency and task *F*(1, 39) = 9.16, *p* = *.*004, *η*_*p*_^2^ = .19, indicating that Simon effects were generally smaller (15.8% – 8.6% = 7.2%) than flanker effects (18.0% – 5.7% = 12.3%). No other effects were significant (with all *p*s *> .*270 and all *η*_*p*_^2^s *< .*032).

For completeness, we also constructed delta plots for the error rates. For each participant and condition (speed-accuracy condition, congruency, task), we rank-ordered the individual RTs (including both correct responses and errors) and then computed the error rate within each bin. Figure A1D shows the mean PEs plotted against the mean RT bins separately for each task and speed-accuracy condition. As can be seen in this figure, the congruency effects in error rates continually decreased with slower responses for all conditions.

## Appendix B

### Additional information regarding DMC model fitting

***DMC fitting procedure***

Following Ulrich et al. (2015), the DMCfun package calculates a cost value for both the percentile RT data (RMSE_RT_) and error data (RMSE_CAF_) with the total cost being a weighted sum of the two:

$$ RMSE={w}_{\mathrm{RT}}\cdotp {RMSE}_{\mathrm{RT}}+{w}_{\mathrm{CAF}}\cdotp {RMSE}_{\mathrm{CAF}} $$

with

$$ {RMSE}_{RT}=\sqrt{\frac{1}{18}\sum \limits_{b=1}^9\sum \limits_{c=1}^2{\left[{Q}^{th;b,c}\hbox{--} {Q}^{ob;b,c}\right]}^2} $$

and

$$ {RMSE}_{CAF}=\sqrt{\frac{1}{10}\sum \limits_{b=1}^5\sum \limits_{c=1}^2{\left[{CAF}^{th;b,c}\hbox{--} {CAF}^{ob;b,c}\right]}^2} $$

where the constants *w*_RT_ and *w*_CAF_ weight the RMSE contributions of CDF and CAF to the overall RMSE. Q_*th;b,c*_ and Q_*ob;b,c*_ are the predicted and observed percentiles of the RT distribution, respectively. The index *b* runs over the nine percentiles of the two congruency conditions (indexed by *c*). CAF_*th;b,c*_ and CAF_*th;b,c*_ are the predicted and observed values of the CAF. Here, the index i runs over the five equally spaced bins and the index *c* again reflects the two congruency conditions. The weight *w*_RT_ was

$$ {\mathit{\mathsf{w}}}_{\mathsf{RT}}=\frac{2\cdot 9}{2\cdot 9+10} $$

and the weight *w*_CAF_ was

$$ {\mathit{\mathsf{w}}}_{\mathsf{CAF}}=\left(\mathsf{1}-{\mathit{\mathsf{w}}}_{\mathsf{RT}}\right)\cdotp \mathsf{1,500} $$

The multiplication factor 1,500 was chosen in order to scale the prediction errors of error rates versus RTs. Specifically, a prediction error of 1% for the CAF corresponds to a prediction error of 0*.*01 *·* 1,500 = 15 ms for the RT quantiles.

The R-package DMCfun offers two fitting procedures to minimize RSMEs by using two other R-packages: First, the R-package optimr (Nash & Varadhan, 2016) which uses the Simplex algorithm (Nelder & Mead, 1965) and second, the R-package DEoptim (Mullen, Ardia, Gil, Windover, & Cline, 2011), which uses the differential evolution algorithm. In the main text, we report the results using the DEoptim fitting procedure to individual data, but we would like to emphasize that similar results were also obtained using the optimr fitting procedure to individual data as well as when applying these fitting procedures to aggregated data.

***Predicted activation functions***

Figure B1 shows the mean activation functions based on simulation results with the best-fitting parameters displayed in Table 1.

## Appendix C

### Additional information regarding ASR model fitting

The activation suppression race (ASR) model of Miller and Schwarz (in press) was developed primarily to explain the RTs in conflict tasks. The model assumes that there is a race between one process suppressing the automatically-extracted distractor-based activation (process A) and another process identifying the relevant stimulus attribute (process B). The durations of these processes are modeled as independent exponentially distributed random variables with rates *α* = 1/*τ*_A_ and *β* = 1/*τ*_B_, respectively. Following the completion of process B, all other processes for responding – that is, decision and motor processes – are modeled as a process C with a normally distributed latency, with the mean and standard deviation of this latency potentially depending on (a) whether the distractor-based activation is excitatory or inhibitory (i.e., congruent versus incongruent trials), and (b) whether or not the distractor-based activation was suppressed before the relevant attribute was identified (i.e., whether process A or B finished first). If A finishes before B in either congruent or incongruent trials, distracting information is suppressed so the distractor-based activation produces no excitation or inhibition. Thus, the duration of process C has mean *μ*_C_ and standard deviation *σ*_C_. If B finishes before A, distractor-based activation does have an effect, so the duration of process C has mean *μ*_C_ + *λ*_exc_ and standard deviation *σ*_Cexc_ in congruent trials and mean *μ*_C_ + *λ*_inh_ and standard deviation *σ*_Cinh_ in incongruent trials, with *λ*_exc_ *<* 0 reflecting facilitation and *λ*_inh_ *>* 0 reflecting inhibition due to the distractor-based activation. Assuming that the relevant and irrelevant stimulus attributes appear simultaneously (i.e., SOA = 0), the probability of B finishing before A is simply $P\left(A>B\right)=\frac{\beta }{\alpha +\beta }$.

The ASR model was fit to the observed individual RT data of the four experimental conditions (i.e., Simon/Eriksen *×* Speed/Accuracy) from each participant by maximum likelihood. The model fit nearly as well with the restrictions *λ*_exc_ = 0 and *σ*_Cexc_ = *σ*_C_, so we report the fits from this somewhat simplified version of the model. Figure C1 shows that the model provides a good fit to the data and a summary of the best-fitting parameters is shown in Table C1. In the following, we report the results of repeated-measures ANOVAs with the two factors task (Eriksen, Simon) and speed-accuracy condition (SP, AC) on the estimated parameter values and on the derived probability of distractor-based activation finishing before central processing starts (i.e., A < B ).

**Table:** Table 2

	Simon task		Eriksen task	
	Speed	Accuracy	Speed	Accuracy
*ASR best-fitting parameters*				
*τ*_A_	65.9	61.3	20.3	40.6
*τ*_B_	47 (4)	76 (6)	50 (3)	78 (8)
*μ*_C_	321 (7)	369 (5)	334 (7)	382 (6)
*σ*_C_	46 (3)	36 (2)	40 (4)	29 (2)
*Λ*inh	63 (7)	62 (20)	142 (55)	283 (117)
*σ*Cinh	46 (7)	29 (8)	76 (15)	43 (6)
*Probability of interference*				
1 – $\frac{\tau \mathrm{B}}{\tau \mathrm{A}+\tau \mathrm{B}}$	0.59 (0.05)	0.51 (0.06)	0.39 (0.06)	0.48 (0.06)

Summary of best-fitting parameters of the Activation Suppression Race model (ASR) (Miller & Schwarz, in press) to the experimental reaction time data of the four subconditions (i.e., Simon/Eriksen × Speed/Accuracy). The parameters τ_A_ and τ_B_ reflect the mean durations of the parallel-running race processes A and B. The parameters μ_C_ and σ_C_ reflect the mean duration and corresponding variability of process C. The parameter λ_inh_ reflects the increase in μ_C_ when B<A in an incongruent trial and the parameter σ_Cinh_ reflects the corresponding variability of this increase. The last row shows the average probability of interference calculated based on the individual values of τ_A_ and τ_B_

Note that the depicted τ_A_ values are medians calculated from the individual condition-specific values, whereas all other parameters are means. Following Miller and Schwarz (in press), median values are more appropriate as summary measure for τ_A_, because the average τ_A_ can be unrealistically inflated by single huge τ_A_ values

Standard errors (SEs) of the means are given in parentheses

As can be seen in Table C1, target features seem to activate responses more quickly with SP compared to AC focus in both conflict tasks (cf. values of *τ*_B_ and *μ*_C_). Indeed, there was a significant main effect of speed-accuracy condition for stimulus identification times (with *τ*_B,SP_ = 49 ms and *τ*_B,AC_ = 78 ms), *F*(1, 31) = 25.62, *p < .*001, *η*_*p*_^2^ = .45 (with all other *p*s *> .*223 and *η*_*p*_^2^s *< .*05). For decision and motor processes, there was a significant main effect of speed-accuracy condition (with *μ*_C,SP_ = 327 ms and *μ*_C,AC_ = 375 ms), *F*(1, 31) = 64.60, *p < .*002, *η*_*p*_^2^ = .68, as well as a significant main effect of task (with *μ*_C,Simon_ = 345 ms and *μ*_C,Eriksen_ = 358 ms) *F*(1, 31) = 11.62, *p* = *.*002, *η*_*p*_^2^ = .27 (with *p* = 967 and *η*_*p*_^2^ *< .*01 for the interaction). The ANOVA on the variability *σ*_C_ of these processes yielded significant main effects of task (with *σ*_C,Simon_ = 41 ms and *σ*_C,Eriksen_ = 35 ms), *F*(1, 31) = 13.28, *p* = *.*001, *η*_*p*_^2^ = .30, and speed-accuracy condition (with *σ*_C,SC_ = 43 ms and *σ*_C,AC_ = 32 ms), *F*(1, 31) = 8.23, *p* = *.*007, *η*_*p*_^2^ = .21, but no interaction (*p* = *.*857, *η*_*p*_^2^ < .01). The ANOVA on the variability *σ*_Cinh_ of these processes when there was interference also yielded significant main effects of task (with *σ*_Cinh,Simon_ = 38 ms and *σ*_Cinh,Eriksen_ = 60 ms), *F*(1, 31) = 6.16, *p* = *.*019, *η*_*p*_^2^ = .17, and speed-accuracy condition (with *σ*_Cinh,SC_ = 61 ms and *σ*_Cinh,AC_ = 36 ms), *F*(1, 31) = 5.94, *p* = *.*021, *η*_*p*_^2^ = .16, (with *p* = *.*408, *η*_*p*_^2^ = .02 for the interaction).^8^

With regard to the strength of inhibition (and hence strength of distractor-based activation), the size of interference *λ*_inh_ seemed generally larger in the Eriksen compared to the Simon task (cf. values of *λ*_inh_), which would fit well to the observed larger flanker versus Simon effects found in the present study. Furthermore, the size of interference seems to increase with AC compared to SP only in the Eriksen but not in the Simon task. However, the ANOVA on *λ*_inh_ yielded neither a significant main effect of task, *p* = *.*074, *η*_*p*_^2^ = .10, nor a significant interaction (*p* = *.*093, *η*_*p*_^2^ = .09, with *p* = *.*137, *η*_*p*_^2^ = .07 for the main effect of speed-accuracy condition). Critically, the estimated values of *τ*_A_ suggest that the time needed for suppressing distractor-based activation was longer in the Simon than in the Eriksen task. Considering now both *τ*_A_ and *τ*_B_, the resulting probabilities of interference P(A > B) would be larger with speed compared to accuracy focus in the Simon task, but smaller with speed compared to accuracy focus in the Eriksen flanker task (cf. values in the last row of Table C1). Indeed, the ANOVA on these mean probabilities of interference revealed a significant interaction, *F*(1, 31) = 4.79, *p* = *.*036, *η*_*p*_^2^ = .13, (with *p* = *.*059, *η*_*p*_^2^ = .11 for the main effect of task and *p* = *.*919, *η*_*p*_^2^ < .01 for the main effect of speed-accuracy condition).

## Footnotes

1 Specifically, this observation was made by inspecting the descriptive values in the speed and accuracy conditions (sometimes collapsing across experiment-specific conditions), because the direct test statistics have not always been reported or have not revealed significant differences. Note also that White, Ratcliff, and Starns (2011) found no significant difference between Eriksen congruency effects with accuracy (70 ms) compared to speed focus (73 ms). Because flankers were displayed vertically in the study by White et al. (2011), it may be possible that this procedural choice changed the relative timing of distractor- and target-based processes relative to horizontally displayed flankers, just as specific stimulus characteristics (e.g., arrows, colored squares) may affect the temporal overlap of these processes and thus modulate their delta plots (cf. Pratte, 2020) and produce differential SAT influences on Eriksen flanker effects (we thank Mathieu Servant for this suggestion).

2 To our knowledge, there have so far been only two independent studies in which the DMC model was successfully fitted to data from a Simon task (Servant et al., 2018) or an Eriksen flanker task (Hedge et al., 2019) with speed-accuracy manipulations.

3 Preregistration including sample size rationale based on the effect size of a pilot experiment (see Appendix A) is available via the OSF at: https://osf.io/g8uk6.

4 We decided to use percentile-averaging to create RT delta plots instead of five equally sized bins as in our pilot work (see Appendix A) due to the planned modeling work. Note, however, that very similar empirical results were also obtained in analyses using five bins.

5 For completeness, we also conducted separate ANOVAs on the two parameters of the gamma density function. The ANOVA on the scaling parameter *τ* revealed a significant main effect of task with smaller values in the Simon (*τ* = 97 ms) than in the Eriksen task (*τ* = 139 ms), *F*(1, 31) = 11.32, *p* = *.*002, *η*_*p*_^2^ = .27, as well as a significant main effect of speed-accuracy condition with smaller values in SP (*τ* = 98 ms) than in AC (*τ* = 138 ms), *F*(1, 31) = 8.16, *p* = *.*008, *η*_*p*_^2^ = .21 (with *p* = *.*442 and *η*_*p*_^2^ = *.*02 for the interaction). Moreover, the ANOVA on the shape parameter *a* yielded also a significant main effect of task, reflecting on average smaller values in the Simon task (*a* = 1.69) than in the Eriksen task (*a* = 2.00). *F*(1, 31) = 16.89, *p < .*001, *η*_*p*_^2^ = .35 (with all other *p*s *> .*114 and *η*_*p*_^2^s *< .*08).
